# Do Parental Comorbidities Affect the Severity of Autism Spectrum Disorder?

**DOI:** 10.7759/cureus.32702

**Published:** 2022-12-19

**Authors:** Hussain Aldera, Ahmed Hilabi, Mohamed R Elzahrani, Moustafa S Alhamadh, Muhannad Q Alqirnas, Reem Alkahtani, Emad Masuadi

**Affiliations:** 1 Department of Basic Medical Sciences, College of Medicine, King Saud bin Abdulaziz University for Health Sciences, Riyadh, SAU; 2 Department of Medicine, College of Medicine, King Saud bin Abdulaziz University for Health Sciences, Riyadh, SAU; 3 Department of Internal Medicine, College of Medicine, King Saud bin Abdulaziz University for Health Sciences, King Abdullah International Medical Research Center, Ministry of National Guard Health Affairs, Riyadh, SAU; 4 Department of Medicine, College of Medicine, King Saud bin Abdulaziz University for Health Sciences, King Abdullah International Medical Research Center, Ministry of National Guard Health Affairs, Riyadh, SAU; 5 Department of Medical Education, College of Medicine, King Saud bin Abdulaziz University for Health Sciences, Riyadh, SAU

**Keywords:** autism severity, autism spectrum disorder (asd), autism treatment evaluation checklist, parental comorbidities, autism

## Abstract

Background

Autism spectrum disorder (ASD) is a group of neurodevelopmental disorders characterized by defective social communication and interaction with a repetitive pattern of monotonous or stereotyped behavior. Although the exact etiology of ASD is unknown, many factors may be implicated in the development of ASD. We aimed to determine the correlation between specific parental factors and Autism Treatment Evaluation Checklist (ATEC) scores.

Methods

This cross-sectional study was conducted at the Prince Nasser Bin Abdulaziz Center for Autism, Autism Center for Excellence, and Academy of Special Education for Autism in Riyadh, Saudi Arabia. We enrolled children diagnosed with ASD and their parents from these centers. Data were collected through self-administered questionnaires to the patients’ parents.

Results

All included children were <18 years old. In total, 71 (92.2%) children were male and six (7.8%) were female. Further, 77 (100%) patients were diagnosed with autistic disorder. Children of consanguineous parents, underweight mothers and obese fathers, mothers with a history of depression during pregnancy, and mothers aged ≥31 years during pregnancy tend to have a higher mean ATEC score. The health domain was the most significantly correlated with ATEC scores, with a Pearson correlation of 0.880. In linear regression analysis, only maternal depression during pregnancy was significantly correlated with ATEC scores.

Conclusion

Our patients had a mean ATEC score of 86.2. The health domain was the most significantly correlated with ATEC scores, with a Pearson correlation of 0.880. Linear regression analysis revealed that consanguinity, parental chronic disease, parental allergy, smoking, drug use during pregnancy, paternal and maternal body mass index (BMI), and sibling number were not significantly correlated with ATEC scores (P=0.701, 0.693, 0.133, 0.874, 0.982, 0.255, 0.778, and 0.502, respectively). However, maternal depression during pregnancy was significantly correlated with ATEC scores (P=0.055).

## Introduction

Autism spectrum disorder (ASD) is a neurodevelopmental disorder characterized by a serious loss of communication and socialization with a pattern of monotonous or stereotyped behavior. According to the Diagnostic and Statistical Manual of Mental Disorders, Fifth Edition (DSM-5), ASD can be categorized as pervasive development disorder, autistic disorder, Asperger’s syndrome, childhood disintegrative disorder, and Rett’s syndrome. Approximately 1% of the population is affected by ASD [1]. In Saudi Arabia, 42,500 confirmed cases of ASD [2] were reported in 2002, a number dramatically higher than that reported worldwide. Since the 1960s, the prevalence of ASD and higher body mass index (BMI) has been dramatically increasing. Thus far, ASD has not been sufficiently studied, and its comorbidities and pathogenesis are unclear.

Some studies have shown that individuals with ASD have the same comorbidities as those with normal neurodevelopment, but their presentation may differ [3]. Furthermore, some studies have shown that some characteristics of ASD may impede the diagnosis of neuropsychiatric comorbid conditions by physicians used to diagnosing disorders with usual presentation and symptoms in normal society [4,5]. Individuals with impairment in social interactions and deficits in speech in addition to ASD are generally unable to explain their issue appropriately and, therefore, unable to explain their physical symptoms adequately. Furthermore, individuals with impairments in sensory processing and ASD may face issues in reporting the location of the pain or any discomfort due to an underlying medical issue [6], which may lead to suboptimal patient care or delayed medical care.

Moreover, it is important to consider the nutritional condition of the parents and their affected offspring. The proportion of child-bearing women is more than one-third of the population [7]. Among them, 9% have prepregnancy diabetes and pregestational diabetes, and an additional 2%-9% develop gestational diabetes during pregnancy [8]. In addition, evidence has shown a correlation between maternal and paternal diabetes and a higher incidence of ASD in children [9]. All subtypes of ASD are weakly associated with maternal obesity, and ASD is associated with increased paternal weight as children of overweight fathers are at risk of developing Asperger’s disorder and ASD [10].

Based on the aforementioned facts and considerations, there is a lack of knowledge on ASD in general and, specifically, on the association between parental comorbidities and ASD severity in children. Hence, we aimed to evaluate the association between these comorbidities and ASD severity.

## Materials and methods

Study design and setting

This cross-sectional study was conducted at Prince Nasser Bin Abdulaziz Center for Autism, Autism Center for Excellence, and Academy of Special Education for Autism in Riyadh, Saudi Arabia. We included children diagnosed with ASD and their parents from these centers. We included all patients with a confirmed ASD according to the DSM-5 criteria and excluded all patients aged >18 years.

Data collection

We collected data through separate self-administered questionnaires on patient demographics, premorbid conditions, and Autism Treatment Evaluation Checklist (ATEC) scores for each domain. Data on parents’ demographics, premorbid conditions, familial contiguity, smoking status, and drug use during pregnancy were also collected. Furthermore, data on siblings with chronic diseases and ASD were collected. We included all patients with a confirmed diagnosis of any of the five types of ASD according to the DSM-5 criteria and excluded all patients aged >18 years. Assuming the prevalence of specific comorbidities in children diagnosed with ASD of 50% and using the Raosoft calculator (Raosoft, Inc., Seattle, WA, USA) set at a 95% confidence interval and 5% margin of error, the sample size was determined to be 369 patients, but we only obtained data from 77 patients and their parents. Informed consent was obtained from the study participants’ guardians prior to study commencement.

Data were collected using the ATEC to measure the association. This tool assesses four domains: speech, consisting of 14 questions; sociability, consisting of 20 questions; cognitive and sensory, consisting of 18 questions; and physical and behavioral health, consisting of 25 questions. A validated Arabic version of the ATEC was used in this study.

The ATEC was developed by Rimland and Edelson [11]. It is easy to use, translate, and complete. This self-administered questionnaire consists of a one-page checklist that is administered to patients’ parents. The ATEC is easily accessible and freely available online, and it does not require any training or previous knowledge of ASD to complete. The ATEC questionnaire is reliable; the Pearson split-half coefficient reliability test on over 1,300 completed ATEC questionnaires revealed a high score of 94 [12]. The validity of the ATEC was reviewed in August 2018, and its efficacy was found to be high [13]. The main goal of the ATEC is to assess the clinical improvement of ASD patients during a particular treatment or intervention. However, recently, there have been multiple published studies that utilized the ATEC score to assess the severity of ASD symptoms [14-16]. The higher the ATEC score, the greater the degree of impairment the patient has from the ASD symptoms. Other than the ATEC, the parental data were gathered using a separate form of multiple questions, one of which was asking if there was a diagnosis of depression during pregnancy or not.

Statistical analysis

The Statistical Package for the Social Sciences (SPSS) version 22 (IBM Corp., Armonk, NY, USA) was used for data analysis. Categorical variables are presented as frequencies and percentages, whereas numerical variables are presented as the mean±standard deviation (SD). A linear regression model was used to test the relationship between the dependent variable ATEC and the other independent variables. Statistical significance was set at P<0.05.

## Results

Patient characteristics

Initially, 369 children diagnosed with ASD according to the DSM-5 criteria and their parents were asked to complete the ATEC questionnaire. Only 77 parents completed the questionnaire, with a response rate of 20%. All included children were <18 years old. In total, 77 (100%) patients had an autistic disorder. Only two patients had siblings with ASD, and only one patient had a sibling with chronic disease (Table 1).

**Table 1:** Demographics of children with ASD and their parents ASD: autism spectrum disorder

		Number	%
Child gender	Male	71	92.2
	Female	6	7.8
Diagnosis	Autistic disorder	64	83.1
	Unknown diagnosis	13	16.9
Sibling with chronic disease	No	76	98.7
	Yes	1	1.3
Sibling with an autism diagnosis	No	74	97.4
	Yes	2	2.6
Mother’s nationality	Non-Saudi	4	5.2
	Saudi	73	94.8
Father’s nationality	Non-Saudi	1	1.3
	Saudi	76	98.7
Parents related	No	45	58.4
	Yes	32	41.6
Maternal body mass index groups	Non-obese	63	81.8
	Obese	14	18.2
Paternal body mass index groups	Non-obese	51	66.2
	Obese	26	33.8
Psychiatry illnesses in parents	No	76	98.7
	Yes	1	1.3
Chronic diseases in parents	No	63	81.8
	Yes	14	18.2
Allergies in parents	No	60	77.9
	Yes	17	22.1
Parents smoke	No	44	57.1
	Yes	33	42.9
Drug use during pregnancy	No	51	66.2
	Yes	26	33.8
Maternal depression diagnosis during pregnancy	No	73	94.8
	Yes	4	5.2
Maternal smoking during pregnancy	No	77	100
	Yes	0	0
Age at pregnancy	≤25	27	35.1
	26-30	24	31.2
	≥31	26	33.8
Number of siblings	0 or 1	16	22.2
	2	13	18.1
	3	18	25.
	4	11	15.3
	≥5	14	19.4

Parent characteristics

Data on parents’ characteristics were obtained through self-administered questionnaires, as mentioned in the Materials & Methods section. Most parents were Saudis, 32 (41.6%) of whom were consanguineous. The prevalence of pregestation underweight, overweight, and obese mothers was 5.2%, 35.1%, and 18.2%, respectively. Further, the prevalence of pregestation underweight, overweight, and obese fathers was 0%, 28.6%, and 33.8%, respectively. Only one parent had a history of psychiatric illness, and 14 (18.2%) had a history of chronic disease. None of the mothers reported active smoking during pregnancy. Four (5.2%) mothers experienced depression during pregnancy. The age of 35.1%, 31.2%, and 33.8% of mothers during pregnancy was ≤25, 26-30, and ≥31 years, respectively, with an average age of 28.5 years. Most parents had three (25%) to four (15.3%) siblings (mean: 3.2 siblings) (Table 1). Children of consanguineous parents, underweight mothers and obese fathers, mothers with a history of depression during pregnancy, and mothers aged ≥31 years during pregnancy tended to have a higher mean ATEC score, but there was no significant difference, except maternal depression during pregnancy (P=0.0286) (Table 2).

**Table 2:** Mean and SD of the ATEC with each parental characteristic SD: standard deviation, ATEC: Autism Treatment Evaluation Checklist

		ATEC	
		Mean	SD
Parents related	No	85.73	30.27
	Yes	86.97	25.17
Maternal body mass index groups	Non-obese	86.14	28.41
	Obese	86.71	27.96
Paternal body mass index groups	Non-obese	83.64	27.84
	Obese	91.35	29.23
Chronic diseases in parents	No	87.17	28.24
	Yes	82.07	28.06
Allergies in parents	No	88.65	27.77
	Yes	77.76	28.4
Parents’ smoking status	No	85.66	29.72
	Yes	87.03	26.19
Drug use during pregnancy	No	85.31	29.55
	Yes	88.08	25.45
Maternal depression diagnosis during pregnancy	No	84.62	27.93
	Yes	116	4.9
Age at pregnancy	≤25	87.56	33.38
	26-30	82.63	22.49
	≥31	88.23	27.51
Number of siblings	0 or 1	83.06	25.09
	2	87.46	28.95
	3	81.39	37.4
	4	98.36	20.08
	≥5	84.07	24.96

Correlation between ATEC scores and autism severity

**Figure 1:**
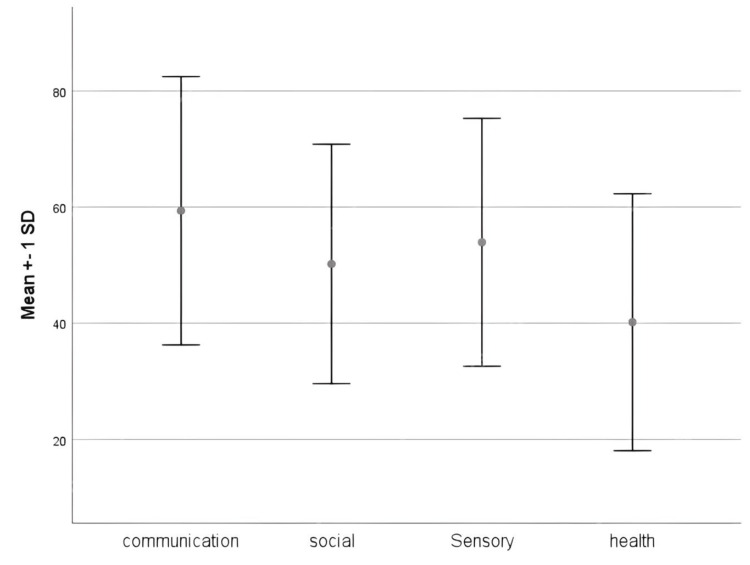
Mean score with the SD of each domain in the ATEC The mean±SD score of each domain of the ATEC is presented as a percentage. SD: standard deviation, ATEC: Autism Treatment Evaluation Checklist

The mean ATEC score was 86.2±28.1 (SD) (range: 12-173). The mean of each domain score is shown in Figure 1. The health domain was the most significantly correlated with the ATEC score, followed by the communication, social, and sensory domains (Table 3).

**Table 3:** Pearson correlation between each domain of the ATEC *Correlation is significant at the 0.05 level (two-tailed). **Correlation is significant at the 0.01 level (two-tailed). ATEC: Autism Treatment Evaluation Checklist

	Communication	Social	Sensory	Health	ATEC
Communication	1				
Social	0.178	1			
Sensory	0.540**	0.019	1		
Health	0.340**	0.457**	0.283*	1	
ATEC	0.631**	0.610**	0.570**	0.880**	1

In linear regression analysis, consanguinity, parental chronic disease, parental allergy, smoking, drug use during pregnancy, paternal and maternal BMI, and sibling number were not significantly correlated with ATEC scores (P=0.701, 0.693, 0.133, 0.874, 0.982, 0.255, 0.778, and 0.502, respectively). However, a history of depression during pregnancy was significantly correlated with ATEC scores (P=0.055) (Table 4).

**Table 4:** Linear regression between dependent variables (ATEC) and other independent variables ATEC: Autism Treatment Evaluation Checklist

Dependent variable (ATEC)	Intercept	P-value	B	95% confidence interval for B	
				Lower bound	Upper bound
Parents related	No	0.701	-3.0	-18.3	12.4
	Yes		0.0		
Chronic diseases in parents	No	0.693	3.7	-14.8	22.1
	Yes (reference)		0.0		
Allergies in parents	No	0.133	13.3	-4.2	30.7
	Yes (reference)		0.0		
Parental smoking	No	0.874	1.2	-14.4	16.9
	Yes (reference)		0.0		
Drug use during pregnancy	No	0.982	0.2	-17.8	18.2
	Yes (reference)		0.0		
Maternal depression diagnosis during pregnancy	No	0.055	-35.5	-71.8	0.8
	Yes (reference)		0.0		
Age at pregnancy	≤25	0.658	-4.5	-24.9	15.8
	26-30	0.948	-0.6	-20.2	18.9
	≥31		0.0		
Maternal body mass index groups	Non-obese	0.778	2.8	-16.7	22.3
	Obese (reference)		0.0		
Paternal body mass index groups	Non-obese	0.255	-9.7	-26.6	7.2
	Obese (reference)		0.0		
Number of siblings	≤2	0.502	-6.2	-24.4	12.1
	>3 (reference)		0.0		

## Discussion

Globally, it is estimated that one in every 160 individuals is diagnosed with ASD. However, in the United States, the rate is estimated to be one in 44 individuals, indicating even higher rates when individuals are well monitored [2,3,17]. Although the exact etiology of ASD is unclear, many factors, including genetics, neuroanatomical abnormalities, prenatal and perinatal factors, parental factors, and environmental factors, have been implicated in the development of ASD [4]. It is unclear whether these factors alone can explain the dramatic increase in the prevalence of ASD [5]. In this study, we explored the association between specific parental factors and ASD severity using the ATEC. To the best of our knowledge, only a few studies have utilized the ATEC scale as an effective tool to assess autism severity [14-16]. However, studies have used the ATEC to assess the response to a particular intervention or treatment.

Owing to the inconsistent findings, the association between parental comorbidities and the risk of ASD in offspring has not been well established. Krakowiak et al. explored whether diabetes, hypertension, and obesity are associated with ASD and developmental delay. They found a higher proportion of diabetes, hypertension, and obesity in mothers of children with ASD, but only obesity showed a significant correlation [18]. Reynolds et al. reported similar results in preterm children [19]. Surén et al. found a significant association between paternal, but not maternal, obesity and the risk of ASD [10]. Li et al. and Sanchez et al. found an association between maternal obesity and the risk of ASD [9,20]. In the present study, parental BMI and chronic disease were not significantly associated with ATEC scores. Because of the small sample size, we decided to only perform linear regression analysis with the ATEC as a whole (Table 3) and not with each domain (communication, social, sensory, and health). This may explain the insignificance of our results. Krakowiak et al. reported significantly worse Mullen Scales of Early Learning and Vineland Adaptive Behavior Scale scores among children with ASD born to mothers with diabetes [18]. This may highlight a possible association between parental obesity and specific chronic diseases, such as diabetes, and impairment in specific functional domains in the population with autism.

Untreated maternal depression during pregnancy has been linked to many deleterious consequences, including low birth weight, preterm labor, and postnatal cognitive and emotional complications, in addition to neuroendocrine and brain function problems [11-13]. Approximately 13% of pregnant women use antidepressants [21]. However, several studies have reported inconsistent findings regarding the association between antidepressant use during pregnancy and the risk of ASD in offspring [21-23]. Boukhris et al. found a significant association between selective serotonin reuptake inhibitor (SSRI) use during the second and third trimesters and the risk of ASD [24]. Moreover, Hagberg et al. have reported that, regardless of antidepressant use, maternal depression increases the risk of ASD [21]. This could be explained by the fact that patients with depression have a higher brain serotonin turnover than healthy individuals [25]. In contrast, Brown et al. found no association between SSRI and serotonin-norepinephrine reuptake inhibitor use and the risk of ASD [26]. Further, Sujan et al. found no significant association between antidepressant use in the first trimester and the risk of ASD [27]. However, Harrington et al. found the opposite results [28]. In our study, we found a significant correlation between a history of depression during pregnancy and ATEC scores (P=0.055). According to our linear regression analysis, having a history of depression during pregnancy would increase the ATEC score by 35.5. Further, higher scores generally indicate more severe ASD or greater functional impairment. Although the P-value is considered borderline, based on our results, we can argue that maternal depression during pregnancy is associated with more severe autism. Further analysis is needed to specify which domain of the ATEC is the most impaired by maternal depression.

Advanced parental age is a risk factor for ASD in offspring. Croen et al. reported a significant association between paternal and maternal age and the risk of ASD in offspring [29]. Additionally, Reynolds et al. concluded that the risk of autism was 5.75% higher in children of parents aged >50 years than in those of parents aged <30 years [19]. However, Reichenberg et al. found an association only between the risk of autism and paternal age [30]. Two meta-analyses on this topic have shown an association between advanced parental age and ASD in offspring [31,32]. This association can be possibly attributed to the mutation of transcription factors, which play a dominant role in gene expression. Furthermore, de novo mutations in male germ cells due to advanced age may be implicated in autism [33]. In our study, we did not find a significant association between age at pregnancy and ATEC scores, which can probably be attributed to the small sample size.

This study has several limitations. First, the study design was descriptive and cross-sectional. Moreover, given that the ATEC is a subjective tool for the assessment of function in the population with autism, it is not highly accurate as parents may overestimate or underestimate their children’s behaviors. Furthermore, some variables included were general or broad. For example, we included chronic diseases in parents and siblings and psychiatric illness in siblings, without specifying the disease itself. Another limitation is that we performed linear regression analysis with the ATEC as a whole and not with each domain (communication, social, sensory, and health). However, we believe that the ATEC is a useful tool to assess autism severity. A multicenter study with a large number of patients focusing on the effect of parental and patient factors on each domain of the ATEC is needed to better guide rehabilitation programs to clarify which domain they should focus on.

## Conclusions

Our patients had a mean ATEC score of 86.2. The health domain was the most significantly correlated with ATEC scores, with a Pearson correlation of 0.880. In linear regression analysis, consanguinity, parental chronic disease, parental allergy, smoking, drug use during pregnancy, paternal and maternal BMI, and sibling number were not significantly correlated with ATEC scores (P=0.701, 0.693, 0.133, 0.874, 0.982, 0.255, 0.778, and 0.502, respectively). However, maternal depression during pregnancy was significantly correlated with ATEC scores (P=0.055).
